# Determining Influenza Virus Shedding at Different Time Points in Madin-Darby Canine Kidney Cell Line

**Published:** 2013-07-02

**Authors:** Asghar Abdoli, Hoorieh Soleimanjahi, Masoumeh Tavassoti Kheiri, Abbas Jamali, Azam Jamaati

**Affiliations:** 1 Department of Virology, Faculty of Medical Science, Tarbiat Modares University, Tehran, Iran; 2 Department of Virology, Influenza Research Laboratory, Pasteur Institute of Iran, Tehran, Iran

**Keywords:** Influenza Virus, Virus Shedding, Endosome, MDCK Cells

## Abstract

**Objective:**

Monitoring of influenza virus shedding and optimization of multiplicities of infection (MOI) is important in the investigation of a virus one step growth cycle and for obtaining a high yield of virus in vaccine development and conventional basic diagnostic methods. However, eluted infectious viruses may still be present immediately after virus inoculation and when cells are washed following virus cultivation which may lead to a false positive virus infectivity assay.

**Materials and Methods:**

In this experimental study, we investigated influenza virus progeny production in Madin-Darby canine kidney (MDCK) cells with five different MOI at determined time points. The results were analyzed by end point titration tests and immunofluorescence assay.

**Results:**

Higher titers of eluted virus were observed following a high MOI inoculation of virus in cell culture. Most probably, this was the result of sialic acid residues from viral hemagglutin in proteins that were cleaved by neuraminidase glycoproteins on the surface of the influenza virus, which promoted viral spread from the host cell to the culture supernatant or during endocytosis, where viruses recycle to the cell surface by recycling endosomes which culminated in virus shedding without replication.

**Conclusion:**

We demonstrated that the pattern of influenza virus progeny production was dose-dependent and not uniform. This production was influenced by several factors, particularly MOI. Understanding the exact features of viral particle propagation has a major impact in producing high virus yields in the development of vaccines. Use of lower MOI (0.01) could result in accurate, precise quantitative assays in virus diagnosis and titration methods.

## Introduction

Influenza viruses are major causes of respiratory tract infection, resulting in significant morbidity and mortality. They are now recognized as a major public health concern and have a significant health and economic burden (1).

Knowledge about the details of influenza virus cultivation within a suitable cell culture is of utmost importance for investigation of its replication and vaccine development. Now a days, much should be done to increase our knowledge about the importance of cell culture based techniques for vaccine development and virus multiplication identification. These techniques should be applied by researchers as before, because they seem to have been forgotten(2).

The tissue culture infective dose 50% (TCID_50_) is one of the basic quantification tests for monitoring *in vitro* influenza virus replication in both research and development (R&D). Although traditional cell culture-based methods are generally slow, labor intensive and time consuming, they are an important, crucial step in viral seed preparation. In the case of influenza virus, the TCID_50_ test is confirmed by the hemagglutination assay (HA), which provides greater reliability. These methods are applicable for further evaluations of influenza virus replication and in optimization of multiplicities of infection (MOI) for virus cultivation in large scales such as vaccine production, determining virus shedding at different time points or *in vitro* evaluation of new antiviral drugs (3-5).

Supernatants are used for quantification of the TCID_50_ in the cultivation of influenza viruses. However, some of the eluted viruses remain detectable, causing false positive test results.

The more the cells are permissive at the virus attachment level and on cell endocytic capacity for internalization, there will be less numbers of eluted viruses in culture supernatants (6, 7).In this study, we have determined the titers of packaged virus at various time points postinfection with different MOI of 1, 0.1, 0.01, 0.001, and 0.0001 in Madin-Darby canine kidney (MDCK) cells.

## Materials and Methods

### Quantification of influenza virus by plaque formation on Madin-Darby canine kidney (MDCK) cells

The MDCK cell lines represent one of the most efficient cell systems for the plaque assay of influenza viruses that are currently available. In this experimental study, we inoculated MDCK cells in six-well culture plates with serial dilutions of the A⁄ Puerto Rico ⁄8 ⁄34 (PR8) virus which was adsorbed in one hour. The inoculums were removed and we washed the cells washed three times with phosphate-buffered saline (PBS). The cell monolayers were covered with a first layer that contained 0.8% cell grade agar (Sigma, St.Louis, MI, USA) in Dulbecco’s modiﬁed Eagle’s medium (DMEM, Gibco, Karlsruhe, Germany), antibiotics (100 IU ⁄ml penicillin and 100 μg ⁄ml streptomycin) without serum, and 2 μg ⁄ml L^-1^-tosylamido- 2-phenylethyl chloromethyl ketone (TPCK)- treated trypsin (Sigma, St.Louis, MI, USA). Plates were incubated for 72 hours and cells overlaid with 1:1000 neutral red (Sigma, St.Louis, MI, USA), 0.8% agar and DMEM for plaque visualization. All culture incubations were performed in a 37˚C, 5% CO_2_ humidiﬁed incubator (8, 9).

### Inoculation of cells with multiplicities of infection (MOI) of viruses

MDCK cells were cultured in DMEM that contained 10% fetal calf serum (FCS, Gibco, Karlsruhe, Germany), 100 IU/ml penicillin and 100 mg/ml streptomycin in six-well plates for 24 hours. Subsequently, cells were washed twice with PBS buffer and inoculated with a ten-fold serial dilution of PR8 virus stock, which resulted in an MOI of 1.0, 0.1, 0.01, 0.001 and 0.0001. After one hour at 37˚C, the cells were washed three times with PBS and supplemented with 3 ml of DMEM that contained 100 IU/ml penicillin and 100 mg/ml streptomycin, without FCS. Finally, 2 μg/ml TPCK-treated trypsin was added to each well (3).

### Time point measurement of virus infectivity titers in Madin-Darby canine kidney (MDCK) cells by 50% tissue culture infective dose (TCID_50_)

We harvested and analyzed culture supernatants for TCID_50_at the following time points (t) post-infection: 1 (immediately after adsorption), 2, 3, 4, 5, 6, 7, 8, 12, 24 and 48 hours. The virus sample was diluted in a 96-well tissue culture plate that contained MDCK. The titration was performed in quadruplicate. In order to confirm TCID_50_ results, the spot HA assay was performed. Virus titers were calculated according to the method of Spearman- Karber (10).

### Hemagglutination assay (HA)

At each time point, 50 μl volumes of the culture supernatants were harvested and diluted in a twofold dilution with PBS.A total of 50 μl of a 0.5% suspension of fowl red blood cells were added to each dilution in a V-shaped microtiter plate. Following gentle agitation, the plates were left undisturbed for 30 minutes at room temperature (RT). The last dilution that showed complete hemagglutination was considered the end point and expressed as hemagglutination units (HAU) per test volume (11, 12).

### Using indirect immunofluorescence for detecting influenza nucleoprotein (NP)

MDCK cells were cultured in 24-well culture plates. Virus inoculation culture supernatants were harvested and monolayer cellswashed three times with PBS, then ﬁxed in 4% formaldehyde for 15 minutes. After washing, 0.02% Triton X-100 (Sigma-Aldrich, Steinheim, Germany) was added and MDCK Cells were incubated for 10 minutes atRT. Antiinfluenza nucleoprotein (anti-NP) monoclonal antibody (Abcam, USA) that contained 1% bovine serum albumin (BSA) was added, and wells were agitated for one hour at RT, washed three times with PBS, then incubated with fluorescein isothiocyanate (FITC)-conjugated secondary antibody anti-mouse IgGthat contained 1% BSA for 1 hour. After washing and drying, cells were observed by inverted immunoﬂuorescence microscopy (Jenus, China) (13, 14).

## Results

### Plaque formation on Madin-Darby canine kidney (MDCK) cells

**Fig 1:**
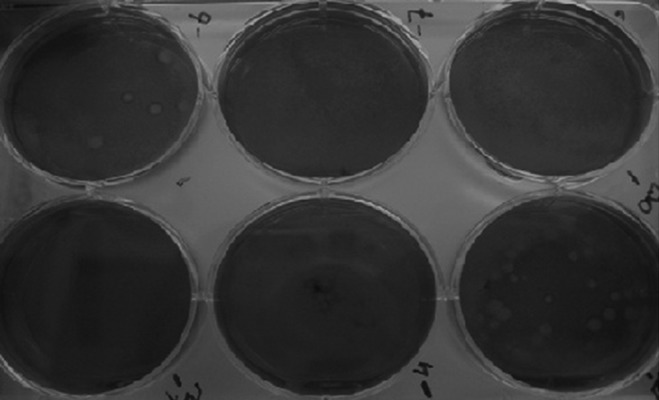
Plaques formed by A⁄Puerto Rico⁄8⁄34 (PR8) viruses on monolayer Madin-Darby canine kidney (MDCK) cells.

Influenza PR8 initiated plaque formation after 48 hours. This resulted in large, flat, indented plaques in the agar (Fig 1). We counted the well that had 15-100 plaques and the viral titer was calculated according to a current microbiology protocol. After adding the second layer of agar that contained 1:1000 neutral red, plates were wrapped in foil to prevent any photodynamic reaction by neutral red (15, 16)

### Determining the highest dilution of viral suspension infectious units using the 50% tissue culture infectious dose (TCID_50_ ) assay

**Fig 2:**
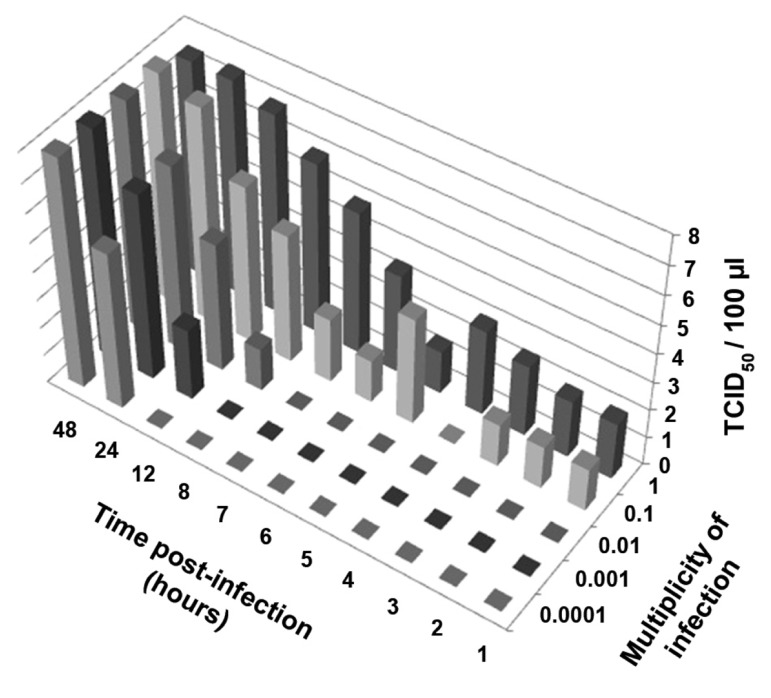
Growth chart of A⁄Puerto Rico⁄8⁄34 (PR8) according to the time point virus infectivity assay for supernatant from Madin-Darby canine kidney (MDCK) cells inoculated at different MOI. The results were expressed asTCID_50_ /100μl.

As can be seen in figure 2, at the MOI of 1 and 0.1, the virus infectivity assay was immediately positive (t1) post-infection. However traces of eluted viruses can create false positives in TCID_50_ analyses. At lower MOI, because the lower titers of viruses have been used for infectivity, eluted viruses are not present. In the current study, virus progeny production was initiated at the following MOI: 0.01 (t8), 0.001 (t12) and 0.0001 (t24).

### Quantification of influenza viruses by hemagglutination assay (HA)

**Fig 3:**
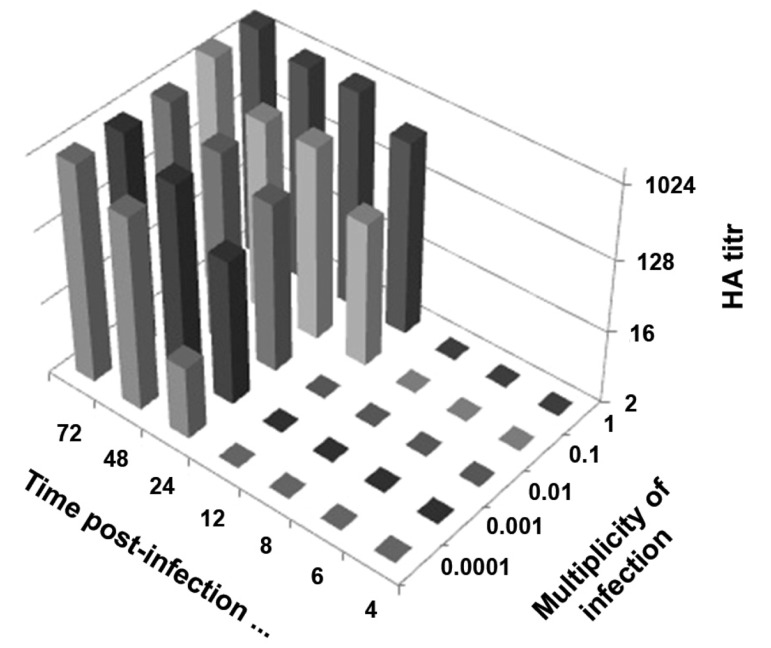
Kinetics of PR8 strain of Influenza A virus replication based on the hemagglutination assay (HA) of Madin-Darby canine kidney (MDCK) cell supernatant, inoculated with different MOI. The results were expressed as HA/50μl.

As shown in figure 3, after 12 hours post-infection, there were 512 HA/50 μl in the MDCK cell supernatant infected at an MOI of 1 and 32 HA/50 μl in the culture supernatant of MDCK cells infected at an MOI of 0.1. At MOI of 0.01, the HA result was 8, at 0.001 it was 128and for 0.0001, the HA test result was 256 units at 24 hours post-infection. Approximately 48 hours post-infection, all MOI reached the same level of HA units; at 72 hours, they reached a peak.

### Immunofluorescence assay

**Fig 4:**
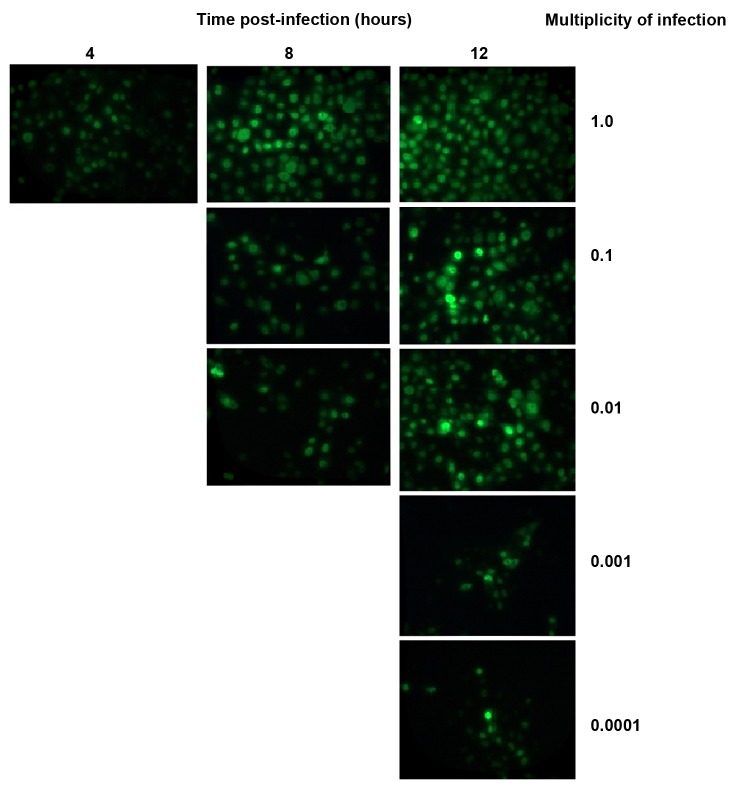
Kinetics of PR8 strain of Influenza A virus replication based on immunofluorescence assay. MDCK cells were inoculated with different MOI and fixed in different times. Then, infected cells observed after incubation with fluorescein labeled antibody specific for the NP protein of influenza viruses (Magnification ×400).

At the MOI of 1, it tookfour hours for a positive result according to the immunofluorescence assay of the influenza virus NP protein (Fig 4). The numbers of NP-expressed cells increased with time elapses, such that after12 hours post-infection; the majority of cells were florescence positive at the MOI of 1. Infected cells with MOI of 0.1 and 0.01 were positive in 8 hours. When MDCK cells were infected at lower MOI (0.001 and 0.0001), the cells were florescence-positive at 12 hours post-infection. The NP protein is an indicator for switching from transcription to replication.

## Discussion

MOI optimization and monitoring of influenza virus shedding has a major impact on high virus yield and outcome of virus seed selection for vaccines. Inﬂuenza viruses are quantiﬁed either by a unit of hemagglutination or by determining infectious units using the TCID_50_ or plaque assays. The interval between inoculation and appearance of detectable virus hemagglutination or TCID_50_ bears an inverse relationship to amount of the inoculated virus (15).

The TCID_50_ assay is a method to gauge the infectious virus in a sample by determining the highest dilution of the sample that can infect 50% of the cells in culture (15). According to TCID_50_ results, at higher MOI (1 and 0.1) eluted viruses remain in the supernatant and do not complete a single virus synthesis cycle, most likely because neuraminidase glycoproteins separate sialic acid residues from viral hemagglutinin proteins. Subsequently,it promotes the spread of the virus from the host cell to culture supernatant(17,18) or the virus returns to the cell surface by recycling endosome which results in virus shedding without progeny production (19).

We detected progeny virus release at 5 and 6 hours post-infection for MOI 1 and 0.1 whereas for MOI of 0.01, 0.001 and 0.0001 they were detectable at 8, 12 and 24 hours after infection, respectively. Henle and Liuhad suggested that the duration of reproductive cycle of influenza virus is 5 to 6 hours based on the length of the interval between the virus inoculation and increasing viral infective titer. Apparently, the elution of some adsorbed virus lead in a substantial amount of asynchronous of infection which confuses the actual virus one-step growth curve (20). Gaush and Smith have shown that a single virus progeny production cycle of influenza virus requires 8 to 10 hours in MDCK cells compared to 20 hours in Chang’s conjunctival cells (21). Since the HA assay is dependent on the amount of hemagglutinin on the surface of inﬂuenza viruses and not on the capability of the virus to replicate, this assay quantiﬁes viral particles apart from their infectivity. Quantitatively, one HAU is equivalent to approximately to 2×10^6^ infectious or noninfectious virus particles/50 μl. At an MOI of 1, 8 hours post-infection is necessary to obtain a positive HA result, whereas for an MOI of 0.1, at 12 hours a positive result is observed (22).

At lower MOI, although HA activity is positive up to 24 hours post-infection, they reach the same HAU within 72 hours. Freymann et al. have demonstrated the appearance of hemagglutin in averaged 8.4, 12.5, 16.3 and 23 hours post-infection when they inoculated 10-day old eggs at MOI of 10^6.5^, 10^5.5^, 10^4.5^ and 10^1.5^ EID_50_, respectively (6).

Portela and Digard (23) in addition to Rimmelzwaan et al. (3) have shown that NP mRNA and NP protein are synthesized during the early infectious phase, which is controlled by the conversion of cRNA into new vRNA. However, Kawakami et al. suggest that both replication and transcription occur simultaneously in the early phase of infection (24). In the current study, we have shown the switch from transcription to translation. We reported that the time to trigger shifting from transcription to replication was around 4 hours post-infection based on NP protein production with an MOI of 1.Replicative intermediate cRNA, stabilized by the newly synthesized NP and viral polymerase, regulates replication (25). Viral matrixM1 protein and NEP/NS2 protein, which is responsible for vRNP nuclear export, inhibit viral transcription at the late phase of infection. Small RNAs generated by the influenza virus (26) and host factors such as BAT1, heat shock protein 90, the minimal chromosome maintenance, Tat-SF1, and DNA dependent RNA polymerase II are involved in influenza vRNA synthesis (27). Experiments have shown that NP protein expression began 4 hours postinfection, whereas at an MOI of 0.1 and 0.01, the time point for NP protein expression was 8 hours post-infection. At an MOI of 0.001, it took 12 hours for the NP protein to be observed by indirect immunofluorescence (Fig 4).

## Conclusion

In this study we compared virus shedding at different time points by determining the TCID5 and A titer. Our data revealed that in higher MOI (1 and 0.1) eluted viruses remained in the supernatant. It took approximately 5 hours for the influenza virus to complete multiplication and produce new progeny at an MOI of 1. The best MOI for influenza virus monitoring in cell culture was 0.01 because no traces of eluted virus were detectable by TCID_50_ and virus progeny production began 8 hours post-infection. At MOI of 0.001 and 0.0001, virus progeny were detectable at 12 and 24 hours after infection, respectively. The optimum time for harvesting the influenza virus has been shown to be 72 hours post-infection due to virus reach the pick of progeny production and the pH changes in cell culture media is not tangible up to 72 hours post-infection.
